# The impact of patient skin colour on diagnostic ability and confidence of medical students

**DOI:** 10.1007/s10459-022-10196-6

**Published:** 2023-03-01

**Authors:** Rebecca V. Dodd, Damir Rafi, Ashlyn A. Stackhouse, Celia A. Brown, Rachel J. Westacott, Karim Meeran, Elizabeth Hughes, Paul Wilkinson, Mark Gurnell, Catherine Swales, Amir H. Sam

**Affiliations:** 1 Imperial College School of Medicine, Imperial College London, London, United Kingdom; 2 Warwick Medical School, University of Warwick, Coventry, United Kingdom; 3 Birmingham Medical School, University of Birmingham, Birmingham, United Kingdom; 4 Health Education England, London, United Kingdom; 5 School of Clinical Medicine, University of Cambridge, Cambridge, United Kingdom; 6 Oxford University Medical School, University of Oxford, Oxford, United Kingdom

**Keywords:** Cognitive processing methods, Diagnostic ability, Diagnostic confidence, Educational resources, Medical education, Mixed methods, Racial bias, Think aloud

## Abstract

Previous literature has explored unconscious racial biases in clinical education and medicine, finding that people with darker skin tones can be underrepresented in learning resources and managed differently in a clinical setting. This study aimed to examine whether patient skin colour can affect the diagnostic ability and confidence of medical students, and their cognitive reasoning processes. We presented students with 12 different clinical presentations on both white skin (WS) and non-white skin (NWS). A think aloud (TA) study was conducted to explore students’ cognitive reasoning processes (n = 8). An online quiz was also conducted where students submitted a diagnosis and confidence level for each clinical presentation (n = 185). In the TA interviews, students used similar levels of information gathering and analytical reasoning for each skin type but appeared to display increased uncertainty and reduced non-analytical reasoning methods for the NWS images compared to the WS images. In the online quiz, students were significantly more likely to accurately diagnose five of the 12 clinical presentations (shingles, cellulitis, Lyme disease, eczema and meningococcal disease) on WS compared to NWS (*p* < 0.01). With regards to students’ confidence, they were significantly more confident diagnosing eight of the 12 clinical presentations (shingles, cellulitis, Lyme disease, eczema, meningococcal disease, urticaria, chickenpox and Kawasaki disease) on WS when compared to NWS (*p* < 0.01). These findings highlight the need to improve teaching resources to include a greater diversity of skin colours exhibiting clinical signs, to improve students’ knowledge and confidence, and ultimately, to avoid patients being misdiagnosed due to the colour of their skin.

## Introduction

There has been growing interest in recent years regarding the potential for unconscious racial biases in clinical medicine. This has been brought into sharp focus by the Black Lives Matter movement (The Lancet, 2020) and the racial discrepancies in mortality rates during the initial phase of the COVID-19 pandemic (Office for National Statistics, 2020). Previous literature has explored aspects of racial bias, to determine whether a patient’s race can affect clinical decision making and management of patients (Dehon et al., 2017; Pezzin et al., 2007; Rathore et al., 2000; Van Ryn & Burke, 2000). A systematic review into the impact of racial bias on clinical decision making, found that despite clinicians having an implicit preference for white people (mostly tested using the Black-White Implicit Association Test), this did not appear to impact their clinical decision making, when examined using case vignettes (Dehon et al., 2017). However, one study demonstrated that a patient’s race can affect the diagnostic tests offered to patients who present with chest pain, with African American patients being offered fewer investigations than non-African Americans (Pezzin et al., 2007). Another demonstrated that physicians tended to perceive African-American patients as less intelligent and less likely to comply with cardiac rehabilitation, post treatment for coronary artery disease, compared to white patients (Van Ryn & Burke, 2000).

Following on from these differences, several studies have reviewed educational resources available for medical students (Adelekun et al., 2020; Louie & Wilkes, 2018), to determine whether these sufficiently prepare students for diagnosing people of all races and ethnicities. A recent study reviewing U.S. editions of popular anatomy textbooks found that while textbook images generally represented the racial distribution of the U.S. population, when skin colour was examined separately, light skin tone was overrepresented and dark skin underrepresented (Louie & Wilkes, 2018). Similarly, an American study of skin phenotypes in dermatology textbooks found limited darker-skin images for some common diseases (e.g. acne, dermatitis), but well represented pictures for some infectious diseases (e.g. syphilis) (Adelekun et al., 2020). This suggests a potential bias as to which conditions are considered to be associated with different skin colours (Adelekun et al., 2020).

To avoid potential bias in medical practice and ensure that students are adequately prepared to deal with diverse patient populations, it is vital that they are exposed to clinical presentations in people of all skin colours. This is especially relevant when considering the cognitive processing methods by which students learn. The dual process framework is a popular structure for describing the cognitive processes used in clinical decision making (Monteiro & Norman, 2013). It comprises two systems: System 1 (non-analytical) being a fast, unconscious system, for example pattern recognition, and System 2 (analytical) a slow process, involving problem solving and applying knowledge (Durning et al., 2015). Whilst perhaps counterintuitive, there is evidence that System 2 processing is not actually the most accurate, with one study showing pattern recognition (System 1 processing), to be around 10 times more effective in reaching an accurate diagnosis (Coderre et al., 2003). In addition to being effective, especially for experts (Coderre et al., 2003), System 1 processing also has a lower cognitive load (Norman, 2009), and thus is more efficient. Given that System 1 processing is automatic, and based on previous memories and experiences (Norman, 2009), clinicians will likely need to have been exposed to a similar case either in their clinical practice, or medical education, to use this method. Whilst medical students as novices will generally be unlikely to use predominantly System 1 clinical reasoning, pattern recognition is the key capability required for making a diagnosis of pathology manifesting as skin disease. Therefore, to ensure accurate diagnoses of all patients, medical students need to be exposed to all skin tones whilst formulating their schema for future pattern recognition.

This study aimed to examine the impact of patient skin colour, on students’ diagnostic ability and confidence in making a diagnosis, when observing clinical presentations on different skin tones. In addition to this, we aimed to explore students’ cognitive reasoning processes, when viewing medical presentations on different skin types, to determine whether a patient’s skin colour has any effect on the processing methods used.

## Methods

### Study design

We used a mixed methods study design, obtaining both qualitative data regarding students’ thought processes, as well as quantitative data regarding their diagnostic ability and confidence. Ethical approval was granted from the Imperial College London Education Ethics Review Process (EERP2021-004a).

### Picture and item selection

**Fig. 1:**
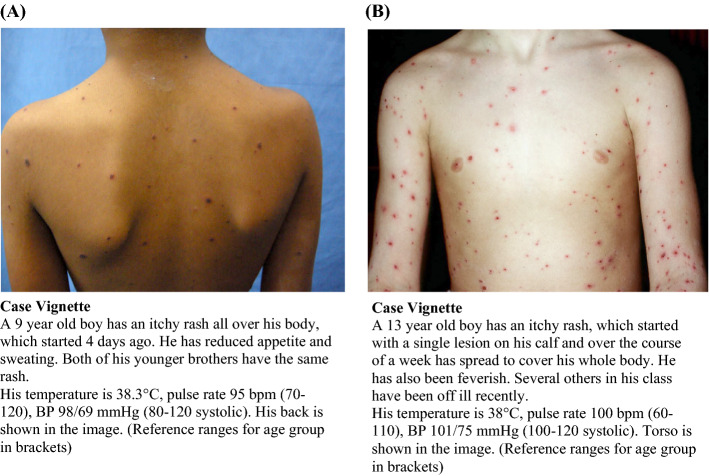
Pictures and case vignettes for chickenpox on NWS (**A**) and WS (**B**). Source for images: ©Waikato District Health Board, used by DermNet New Zealand with permission (DermNet New Zealand Trust)

The Imperial College School of Medicine (ICSM) undergraduate programme is six years in length, with year 5 being the penultimate year, where students have clinical placements in obstetrics and gynaecology, paediatrics, psychiatry, infectious diseases, oncology, general practice and dermatology. We collected 24 medical photographs of the following 12 clinical conditions and signs, relevant to their curriculum: Shingles, Kawasaki disease, cellulitis, pityriasis versicolour, Lyme disease, central cyanosis, eczema, urticaria, chickenpox, meningococcal disease, jaundice and Henoch-Schönlein purpura. For each of these clinical presentations, we used one image of the condition on white skin (WS) and one on non-white skin (NWS), using pictures that matched in body part and quality as closely as possible. The NWS images included Black and Asian ethnic groups, with a range of gradations of skin tone. We gained consent, or licence where applicable, from the relevant source for each picture used in the study. Once the picture bank was complete, a short case vignette was written for each picture by the researchers, based at ICSM. The pictures and vignettes were reviewed for quality and consistency by a further clinician, outside of ICSM. As an example, the NWS and WS pictures and vignettes used for chickenpox are illustrated in Fig. 1.

### Data collection

#### Qualitative approach: think aloud interviews

Year 5 medical students at ICSM were invited via lecture ‘shout-out’s’ and the student newsletter to take part in a Think Aloud (TA) interview. They were told that the study aimed to evaluate students’ diagnostic ability and certainty, along with ascertaining how they cognitively processed different clinical scenarios. To mitigate bias against knowledge of the study aims, the participants were not told that their responses to different skin tones was a key factor being analysed. TA interviews were conducted online via video calls by one of three researchers. To ensure consistency between the TA interviews, the researchers underwent a training session and used a common script. Each TA interview began with a warm-up exercise, where participants were shown two scenarios and a selection of faces showing different emotions and were asked which face fitted best with each scenario. This was followed by participants being shown the 24 pictures of clinical conditions and signs, with their associated case vignettes. The TA component was carried out concurrently, with participants asked to ‘think aloud’ as they interpreted each picture/ case, and they were also asked to suggest a confidence rating (1: ‘complete guess’ to 10: ‘absolutely sure’), depending on how confident they were in their diagnosis.

Once the pictures and vignettes had been shown, participants were asked whether they felt equally confident identifying medical conditions on people of all skin colours, if there was a difference, why they thought that might be and how they thought their confidence might be improved. This was followed by a period of debrief, where participants would ask any questions and find out the correct diagnoses. The interviews were digitally recorded and transcribed.

#### Quantitative approach: quiz

Year 5 medical students at ICSM were invited via their student notices to take part in the quiz as a revision session. Participants that had not yet started their two-week dermatology rotation and those who had already done a TA interview, could take part but had their data excluded from analysis. Similarly, students could take part in the quiz but opt not to have their data used for analysis. Again, details of the skin colour element to the study were not given initially, however this was explained at the end of the session, alongside the correct answers. The quiz took place virtually via a video link, using a cloud-based tool (Mentimeter), whereby a researcher clicked through the series of pictures and questions, and participants submitted their diagnoses and confidence levels (1–10) in real time, using personal digital devices. Students were given up to 60 seconds to answer each question.

### Data analysis

#### Qualitative approach: think aloud interviews

TA interview transcripts were analysed thematically, by inductive coding, using the software Dedoose. During this process, excerpts (pieces of text) from the transcripts were coded to one or more codes. Therefore, the same code could have been applied multiple times within an individual’s response to each question. Similar to previous literature (Sam et al., 2021; Surry et al., 2017), we performed a collaborative analysis of the data, whereby three researchers analysed using a constant comparative approach, re-evaluating previous transcripts when new codes were found. Saturation was achieved after six interviews, with no new codes being required in the last two transcripts. Once all the coding was complete, the three researchers then re-reviewed all the transcripts with the completed codebook to check for consistency and organised the codes into a hierarchical framework, with a set of overarching themes. For each theme, the percentage of excerpts for WS and NWS were calculated as the number of excerpts relating to each skin type, divided by the total number of excerpts for that theme (N).

#### Statistical analysis: quiz

Answers submitted in the quiz were marked by three researchers collaboratively, with care taken to ensure answers were marked consistently across different skin types. Answers which did not match the predetermined list of correct answers were discussed amongst the three researchers. When an alternative answer or spelling mistake was allowed in one instance, this was applied consistently across the quiz. Data management and analysis were conducted using STATA V17.

To examine for a difference in diagnostic accuracy between WS and NWS pictures, matched pair odds ratios (OR) were calculated for each condition. Participants’ answers were only included when they had submitted answers for both the WS and NWS pictures for each condition. Odds ratios were calculated as the number of students who got the NWS picture correct and WS incorrect divided by the number who got WS correct and NWS incorrect, meaning only participants in each of the two opposing groups (WS correct + NWS incorrect and WS incorrect + NWS correct) were included in analysis. McNemar significance testing was carried out for the OR for each condition, with a critical *p* value of 0.01 used.

A total paired difference score for diagnostic ability was calculated for each student, as the sum of the difference score for each condition, for that student. For each student, each condition was given a difference score of either 1 if WS correct and NWS incorrect, 0 if (a) both WS and NWS correct, (b) both WS and NWS incorrect or (c) only one skin type answered or − 1 if WS incorrect and NWS correct. The possible range in total difference scores was therefore − 12 to + 12. A one sample t-test was used to test the null hypothesis that the mean total paired difference score across participants was zero.

When analysing participants’ confidence scores (1–10 for each condition), values were only taken as meaningful and used in analysis if a diagnosis had also been submitted alongside the confidence rating for each picture. Paired differences were calculated for each participant from confidence scores for each skin type (WS-NWS), where participants had answered questions for both skin types displaying each given condition. One-sample Wilcoxon signed-rank tests were carried out for each condition, to determine whether confidence scores were statistically significantly different for WS and NWS pictures, again with a critical *p* value of 0.01 being used.

## Results

### Qualitative approach

#### Participants

A total of eight year 5 students from ICSM participated in TA interviews, which took place between January and March 2021. Participants were aged 22–25 years, six females and two males, with two describing their ethnicity as White, five Asian and one Mixed ethnicity.

#### Codes and themes

Initial analysis produced 38 total codes across 7 themes, which are displayed in Table 1. The seven themes identified were as follows: Non-analytical and analytical reasoning, information gathering, uncertainty, attitudes and emotions, skin colour related comments and noticing/ comparing repeats. Example excerpts from participants relating to each theme are also included in Table 1.

**Table 1:** Table showing the 38 codes identified, separated into 7 broad themes, with example excerpts from participants for each theme

Theme	Codes	Example excerpts (Related code(s) for each theme)
Non-analytical reasoning	Intuition Familiarity Pattern recognition Spot diagnosis Real world experience Recalling information Recognising red flags and buzzwords	WS—“…think these are café au lait spots, because I have seen, I think, a similar picture before.” (Familiarity, spot diagnosis) NWS—“I think that’s what they call angioedema. This is kind of bias because I saw a kid with this last week, that looked similar.” (Familiarity, real world experience) WS—“So, just reading the text. So, he’s 20 years old with a rash, headache and photophobia. So, immediately I’m thinking, could this be meningitis because of [sniffs] this, sort of, triad. And also, given his age.” (Pattern recognition) NWS—“So I guess warm to the touch tends to be, like, a buzzword for cellulitis.” (Pattern recognition, recognising red flags and buzzwords)
Analytical reasoning	Applying knowledge Linking information Deductive reasoning Demographic reasoning Problem solving Process of elimination Deciding between differentials Reasoning based on anatomy Reasoning based on geography Case-picture mismatch Picture-expectation mismatch	WS—“So, the fact that several others in his class have been having it makes me think infection again, also the fact that he’s got a fever.” (Deductive reasoning, linking information) NWS—“And he’s recently been starting a course of… Okay, so maybe it’s a reaction, like a hives-type of reaction to the new medication he’s been started on.” (Problem solving) NWS—“Well, first thing is, this is bilateral, so I’m not thinking cellulitis as much. It's also a little bit more spread out, so I’m thinking, again, that this might be some sort of thrombotic or, like, vascular condition. [Hesitates].” (Deciding between differentials, deductive reasoning) WS—“And given the history, that's inflamed and itchy and, also, her asthma, it makes me think eczema, really. So, I guess it’s eczema.” (Linking information)
Information gathering	Describing rash Describing case vignette Needing more information	WS—“The picture looks like a type of pigmented macular lesions over his torso…” (Describing rash) NWS—“Okay, so the vignette doesn’t help me, apart from the fact that it’s itchy.” (Describing case vignette, needing more information) WS—“Scaly rash and he’s not been unwell. For over the last few weeks, he had some travel history. Otherwise, his observations look very normal.” (Describing case vignette)
Uncertainty	Guessing Hesitancy Doubting Difficulty describing rash Difficulty linking information Inability to reach a confident diagnosis Lack of experience Difficulty in recall Trouble interpreting a 2D Image Relying on case	NWS—“I feel like it's something that I should know but I just don't [laughs]. Okay.” (Difficulty in recall) WS—“So, they don’t seem to have any problems with the breathing yet which makes me less confident about my diagnosis.” (Difficulty linking information, doubting) WS—“This is going to be guesswork on my part [laughs].” (Guessing) NWS—“I’ve not seen anything like this before.” (Lack of experience) WS—“I haven’t actually seen a rash for Kawasaki disease before. So, this is more based on the case. [Hesitates] but from what I’ve seen it described as, it’s just like a widespread maculopapular rash, which is what the picture looks like. So, I’ll go with Kawasaki disease.” (Relying on case, lack of experience, hesitancy)
Attitudes and emotions	High confidence Lack of confidence Laughing (emotional response) Shock (emotional response)	NWS—“[Long pause] Yes [hesitates] [laughs], wait, let me… Must I just choose one [laughs]?” (Laughing (emotional response)) WS—“Oh god, that looks horrific.” (Shock (emotional response))
Skin colour related comments	Comments about skin colour	NWS—“That makes me think of, is it acanthosis nigricans? That, when it’s in a crease and it’s darker. But again, it’s the darker skin tone, it’s kind of difficult to tell.” (Comments about skin colour) NWS—“like hyper pigmented lesions or like maybe it’s red, I can’t quite tell from the skin colour.” (Comments about skin colour) WS—“… based off the history, I’m thinking chicken pox, but it’s, [hesitates], I guess I’m not used to, I haven’t actually seen, [hesitates], chicken pox on such light skin that’s, [hesitates], that red.” (Comments about skin colour)
Noticing/comparing repeats	Comparing between repeats Recognition of repeats	WS—“So, this is probably chickenpox again.” (Recognition of repeats) NWS—“I think that this is likely to be an eczema as well but presenting in a girl with darker skin.” (Recognition of repeats)

**Fig. 2:**
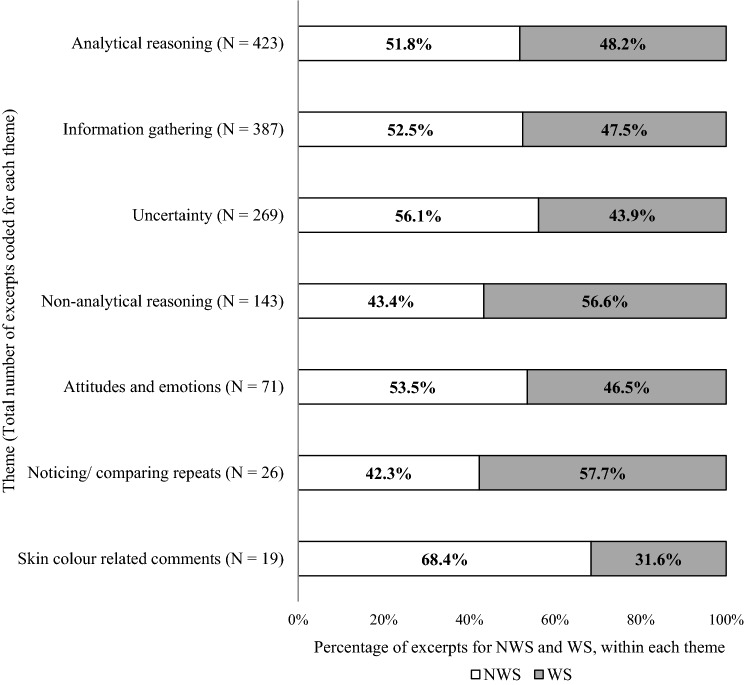
Percentage of excerpts for NWS and WS, within each theme. N = Number of total excerpts coded for each theme

All participants went through a process of information gathering during the TA interview and combinations of analytical and non-analytical reasoning methods. As shown in Fig. 2, the non-analytical reasoning codes had less assigned excerpts from participants viewing NWS pictures and vignettes as compared to WS (62 (43.4%) compared to 81 (56.6%) respectively), however, for analytical reasoning methods, this difference was less apparent (219 (51.8%) coded to NWS compared to 204 (48.2%) for WS pictures). More excerpts relating to comments about skin colour in the clinical picture were coded when participants were viewing the NWS pictures (13 (68.4%)) compared to the WS picture (6 (31.6%)) (Fig. 2). All participants experienced periods of uncertainty; however, more excerpts were assigned to the uncertainty codes when participants viewed NWS pictures and vignettes (151 (56.1%)) compared to WS (118 (43.9%)) (Fig. 2).

#### Think aloud interviews: final questions

Of the eight think aloud interviews conducted, seven participants reported not feeling equally confident identifying medical conditions on people of all skin colours, with the remaining participant reporting they felt similarly confident because “…even if you have the same skin colour, you can come up with the same rash differently”. Participants described a variety of reasons for the difference in their confidence when diagnosing medical conditions on people of all skin colours, including:

- Difficulty in seeing skin changes on darker skin:

“So, a lot of the conditions you’d pick up on erythema, or you’d pick up on skin being darker or paler. But I feel like on darker skin tones, sometimes it’s the opposite, and sometimes you just don’t notice the erythema.”

- Lack of exposure on dermatology rotations:

“I think it’s in terms of exposure. [Hesitates] probably especially for [hesitates] my year group and the year group above, because our dermatology placements have been quite impacted by COVID. And also they’re not that long, and they’re mostly in the outpatient setting.”

- Lack of exposure of images of dark skin types in textbooks/ educational resources:

“So, I think in dermatology textbooks and in literature, mainly from what we’ve seen it’s all about lighter skin tones that are more documented and yeah… So, I think the lack of representation of darker skin in the textbooks can be detrimental when it comes to like education and when… What the rashes may look like on darker skin tones.”

- Ethnically diverse presentations only being shown in resources in certain circumstances:

“So, I think that, especially in, like, medical school textbooks and online resources, we often only see images of, kind of, white Caucasian people with skin conditions, unless it’s something that’s very … Sorry [laughs], just trying to think of the right word. Unless it’s a [hesitates], skin condition that we only see in a certain, like, ethnic group, for example.”

Participants described a variety of possibilities for how their confidence might be improved, when diagnosing medical conditions on people of all skin colours. These included:

- Increased exposure to skin conditions on all skin colours in medical teaching:

“Probably [hesitates] just more experience and more practice, and in terms of when we get taught about skin conditions. Like in an ideal world, they could show it on as many different skin colours as possible, just to highlight the differences. [Hesitates] but I know that’s probably not super practical.”

- Pairing images of different skin types during teaching, to demonstrate different presentations:

“I think every time I’m shown a diagnosis on a lighter skin tone, if I was shown the exact same diagnosis on a darker skin tone.”

- Increased studying:

“So, I think it’s a bit of a mixture. I think, on one hand, more revision on my own part would improve some of the confidence over some of the presentations.”

- Increased attention when examining patients:

“And also I guess just being prompted to pay more attention for things on darker skin tones. For example if a child came in, usually you just give them a once-over looking for rashes, or you’d ask the parent. I guess the parent would be a better idea, because they’re more likely to know what their child’s skin looked like normally.”

- Questioning first instinct/diagnosis:

“So, yes, I like this, like, rapid-fire thing, but also thinking, can I just do a spot diagnosis and can I then question myself, because, like, sometimes I realise what I thought was completely different to what the history suggested. [Hesitates]. So then, just understanding that, yes, it is, [stammers], you can get to a point where you’re quite good at spotting things, but also you need to always have that voice at the back of your head, saying, [hesitates], just double check, you might be wrong, you might have missed something.”

### Quantitative approach

#### Participants

From a total of 309 year 5 medical students at ICSM, 254 (82.2%) joined the online quiz session, which occurred in April 2021. From these, 221 consented to their data being included, 16 did not consent and 17 did not answer the question about consent, so these latter 33 were excluded from analysis. From the 221 who consented, five students had already done a TA interview and 22 had not yet started their dermatology rotation, therefore these participants were also excluded from data analysis, along with those who did not answer the questions relating to TA interviews and placements (eight participants). One participant did not submit any answers, therefore was also removed. A total of 185 participants were therefore included in data analysis (72.8% of students who joined the session and 59.9% of year 5 students). From the participants who chose to disclose their demographics, 87 were female and 86 were male (49.7% male), aged between 22 and 30 years (Median = 23, Interquartile range (IQR) = 1). Sixty-two participants described their ethnicity as White (35.6%), and 112 as either Asian, Black, mixed/ multiple ethnic groups, or other ethnic groups (46.6% Asian, 5.2% Black, 5.2% mixed ethic groups and 7.5% other ethnic groups). The remaining participants did not disclose their demographics.

#### Diagnostic ability

**Fig. 3:**
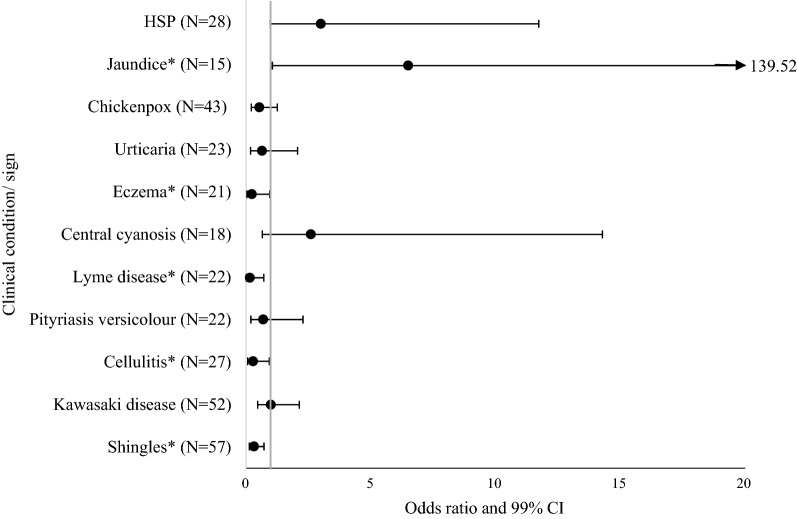
Matched pairs odds ratios and 99% confidence intervals for diagnostic ability by condition. The vertical line denotes an OR of 1. OR values can be interpreted as follows; 1 = No difference in diagnostic ability of WS picture compared to NWS, < 1 = Increased diagnostic ability of WS picture compared to NWS, and > 1 = Increased diagnostic ability of NWS picture compared to WS. Meningococcal disease was excluded, due to a 0 value in one of the cells of the contingency table meaning an OR could not be calculated. N = Number of participants across opposing groups (WS correct + NWS incorrect and WS incorrect + NWS correct). * Statistically significant at level *p* < 0.01

As demonstrated in Fig. 3, by the majority of ORs falling to the left of the vertical line (OR = 1), participants had a greater ability when diagnosing clinical conditions and signs on WS as opposed to NWS (OR < 1), with the exceptions being: Kawasaki disease, jaundice, central cyanosis and HSP. There was a statistically significant improvement in students’ ability to diagnose shingles, cellulitis, Lyme disease, eczema and meningococcal disease from the WS picture, when compared to the NWS picture. The OR for meningococcal disease could not be calculated, as 34 participants correctly diagnosed only the WS picture, but none correctly diagnosed only the NWS picture (Appendix 1). For jaundice the reverse was true, with there being a statistically significant increased ability for participants diagnosing the NWS picture compared to the WS picture. There were no statistically significant differences in diagnostic ability for chickenpox, HSP, urticaria, central cyanosis, pityriasis versicolour and Kawasaki disease. It should be noted that jaundice, central cyanosis and HSP had the widest 99% CI ranges (Fig. 3). Values of each OR, along with McNemar significance testing for each condition are shown in Appendix 1.

A one sample T test against the null hypothesis value of 0 showed that overall, participants had increased diagnostic accuracy when viewing the WS pictures compared to the NWS pictures (Mean total paired difference score (M) = 0.52, standard deviation (SD) = 1.49, t(184) = 4.74, *p* < 0.001). Overall, a small to medium Cohen’s d effect size was demonstrated (d = 0.35).

#### Confidence in diagnostic ability

**Fig. 4:**
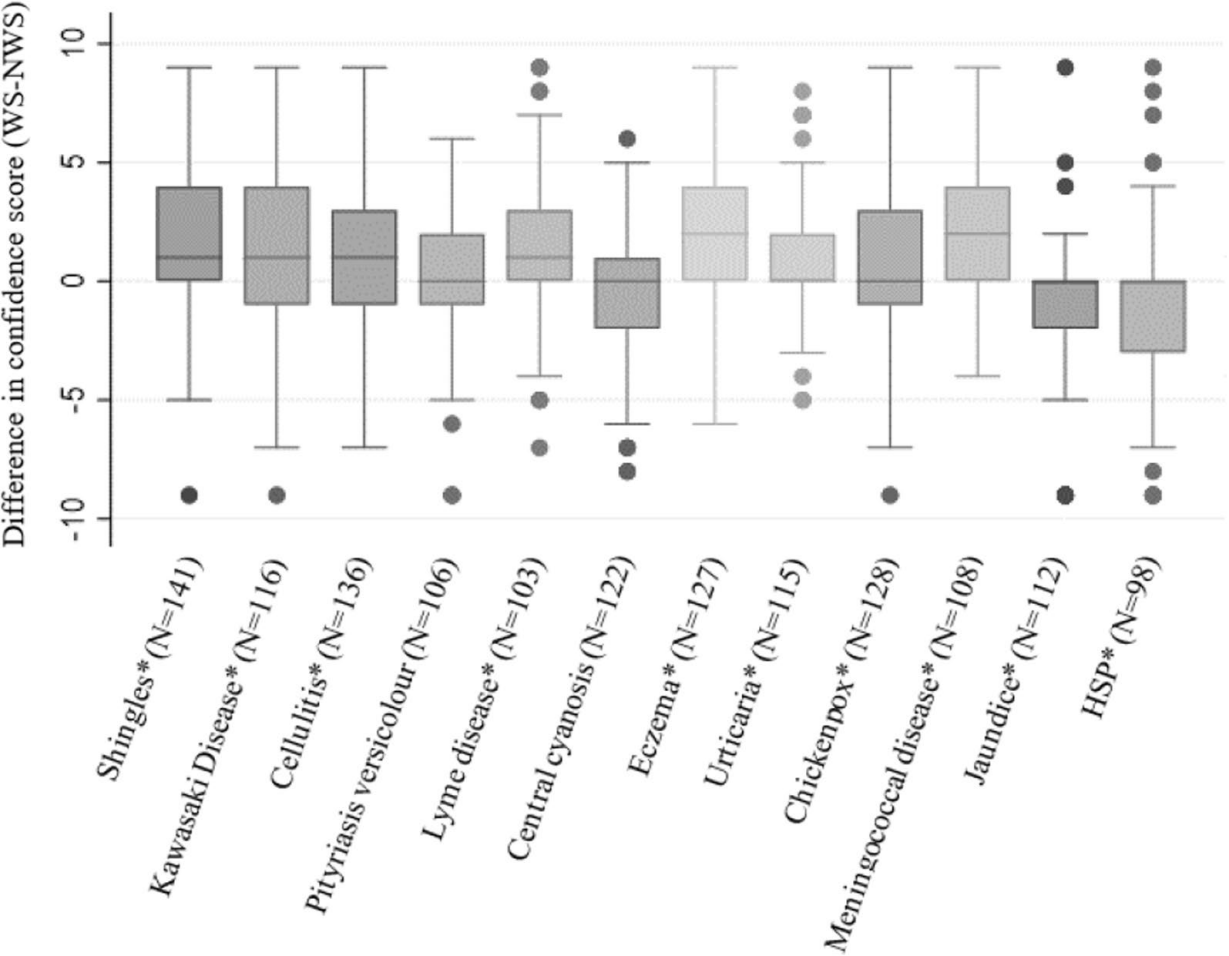
Boxplot of paired difference in confidence scores. Values for paired difference in confidence scores can be interpreted as follows; 0 = no difference, > 0 = Increased confidence diagnosing conditions on WS, < 0 = increased confidence diagnosing conditions on NWS. N = Number of participants who submitted confidence ratings alongside a diagnosis, for both the WS and NWS picture of each clinical condition/sign. * Statistically significant at level *p* < 0.01

The median and IQR of the paired difference in confidence scores (WS–NWS) for each condition is represented in Fig. 4. This shows a median for each condition of either 0 or above, indicating a tendency for participants to be either similarly confident or less confident diagnosing skin conditions on NWS compared to WS (Fig. 4). A Wilcoxon signed-rank test showed that participants were statistically significantly more confident diagnosing shingles, Kawasaki disease, cellulitis, Lyme disease, eczema, urticaria, chickenpox and meningococcal disease on WS compared to NWS (Fig. 4, Appendix 2). However, participants were statistically significantly more confident when diagnosing HSP and jaundice on NWS when compared to WS (Fig. 4 and Appendix 2). There were no statistically significant differences in confidence ratings for Pityriasis versicolour or central cyanosis.

## Discussion

This study set out to determine whether medical students’ diagnostic ability and confidence for a range of clinical conditions and signs were affected by patients’ skin colour, by using photographs and case vignettes of clinical presentations on WS and NWS. In addition, we aimed to explore whether students’ cognitive processing methods differed, depending on the skin colour depicted in the clinical image. It is of paramount importance to understand whether students are being adequately prepared for diagnosing medical conditions accurately in patients of all skin colours.

Students were statistically significantly more capable of diagnosing several common clinical presentations (shingles, cellulitis, Lyme disease, eczema and meningococcal disease) on WS compared to NWS. For all these presentations, they were also significantly more confident when diagnosing the condition on WS, along with also being more confident diagnosing urticaria, chickenpox and Kawasaki disease on WS when compared to NWS. It should be noted that the findings for diagnostic ability are an objective measure of test score, whereas the confidence measures were self-ratings, therefore likely more open to subject bias. This finding is mirrored in data from the TA interviews, whereby seven out of the eight participants reported they were not equally confident at diagnosing medical conditions on people of all skin colours. The clinical presentations of jaundice, central cyanosis and HSP however, did not follow the same trend. For jaundice, students were significantly more accurate and confident diagnosing the NWS image, although in this study the diagnosis was based on looking at changes in the sclera rather than a skin change. Whilst not significant, similar findings were observed for central cyanosis, with one possible explanation being that the patient depicted in the NWS photograph of cyanosis was of Southeast Asian origin and had paler skin than the other NWS examples in the study. However, this does not explain why students were more able to diagnose central cyanosis on this patient compared to the patient with white skin. For HSP, students tended to be more accurate diagnosing the NWS picture compared to WS, and were more confident diagnosing the NWS picture, although only the latter achieved statistical significance. There is not a clear explanation to describe the difference demonstrated for HSP.

Considering the TA interviews, whilst all students demonstrated periods of information gathering and analytical reasoning when deciding upon a diagnosis for both skin types, there were some notable differences. Students seemed to express more uncertainty when viewing the NWS pictures, and used non-analytical reasoning (e.g. pattern recognition/ spot diagnosis) more frequently when viewing the WS pictures. The latter is unsurprising, given that non-analytical reasoning requires a level of familiarity and previous experience (Eva, 2005; Norman, 2009) and images of skin conditions on darker skin tones can be underrepresented in medical resources (Adelekun et al., 2020; Louie & Wilkes, 2018). Whilst analytical cognitive processing methods are preferable for medical students, given their lack of substantial experience (Eva, 2005), this study’s findings do support the assumption that students are less familiar with clinical presentations on patients with NWS. This is a cause for concern, as a lack of familiarity with medical conditions on all skin types has the potential to result in patients with darker skin being misdiagnosed or underdiagnosed at a higher rate than those with white skin. However, the implications of this uncertainty should be further investigated, as it is not inconceivable that medical professionals may still come to the correct diagnosis by recognising their uncertainty and seeking further advice. Additionally, of all the identified themes from the TA interviews, the one with the fewest coded excerpts was skin colour related comments. This may indicate that students were not consciously aware of the potential differences and challenges when diagnosing patients of various skin colours.

The TA technique is widely regarded as a way to capture information on cognitive processing in real time and avoid retrospective bias (Birch & Whitehead, 2020). Similar to previous literature (Lundgrén-Laine & Salanterä, 2010), participants took part in a warm-up session to practise the process of ‘thinking-aloud’. The concurrent nature of the TA interviews in this study avoids memory decay (Birch & Whitehead, 2020), however care was taken by the researchers not to interrupt the participants during the tasks and risk causing distortion as previous literature suggests (Burbach et al., 2015), but instead provided gentle prompts such as “keep talking”, during periods of quiet.

The questions asked at the end of the TA interviews add a richness to the data gathered and shed light on possible areas for improvement in medical education, as suggested by the students who participated. Students reported they would benefit from increased exposure to clinical conditions on NWS in textbooks, learning resources, teaching and on clinical placements.

There were several limitations to this study, the first being the limited participant sample size in both the qualitative and quantitative approaches. Whilst sample sizes when using the TA methodology are usually small (Lundgrén-Laine & Salanterä, 2010) and saturation was achieved after coding six transcripts, it should be acknowledged that this is a limited sample size from which to draw any major conclusions. Additionally, the limited sample size did not permit evaluation of the impact of the participants’ own demographics on their responses. Similarly, for the quiz element, complete data for analysis was reduced, due to some students not submitting answers for all questions. This was partially due to the voluntary nature of participation and that the quiz was formative. The study also only had participants from a single medical school. This was a strength in that students were likely to have had very similar teaching and learning experiences but may not be representative of other student cohorts nationally or internationally. It should also be acknowledged that this study took place in 2021 during the worldwide Covid-19 pandemic, a unique time in both medical education and clinical practice.

One further limitation of this study was the lack of images that were available for use in constructing the vignettes for this study, especially for the NWS clinical pictures; this itself illustrates the problem students face when trying to educate themselves about clinical presentations in patients with different skin tones.

## Conclusion

The findings of this study provide insight into the question of whether patient skin colour may affect medical students’ diagnostic ability and confidence. Whilst this pilot study is relatively small in participant numbers, it has broad implications for medical education. This study demonstrated that medical students lacked confidence diagnosing several common clinical presentations in people with NWS and were also less accurate when diagnosing several presentations on NWS, compared to WS. This suggests that medical educators and teaching resources are not adequately preparing students for managing an ethnically diverse patient population. If students do not have exposure to signs on a wide variety of skin tones, their ability to generate schema to allow non-analytical (system 1) thinking will be compromised. Medical students have previously expressed concern about the lack of diverse representation in their education (Nolen, 2020), and it is vital that prompt action is taken to address this.

Medical educators need to include diverse presentations in teaching, showing how diseases may manifest on as many skin types as possible, and support students in where they can access further images. One notable resource is VisualDx (https://www.visualdx.com/), which contains medical images across all skin types and pigmentations, but is not freely available to all. Several clinician, student and patient led groups however, are creating free image banks of clinical presentations on a diverse range of patient skin types, such as Black and Brown Skin (https://www.blackandbrownskin.co.uk/) Brown Skin Matters (https://brownskinmatters.com/) and Skin Deep: A DFTB Project (https://dftbskindeep.com/). Improved teaching on the diversity of presentations in different skin tones is necessary to provide students with the tools and resources they require to serve diverse patient populations and to avoid patients being misdiagnosed due to the colour of their skin.

## Appendix 1

See Table

**Table 2:** Matched pair odds ratios for diagnostic ability by condition

	OR^a^	Upper 99% CI	Lower 99% CI	N^b^	McNemar’s chi^2^	*P* value^c^
Shingles	0.33	0.72	0.13	57	14.75	0.0002
Kawasaki disease	1	2.13	0.47	52	0	1
Cellulitis	0.29	0.93	0.06	27	8.33	0.0059
Pityriasis versicolour	0.69	2.28	0.19	22	0.73	0.5235
Lyme disease	0.16	0.71	0.02	22	11.64	0.0009
Central cyanosis	2.6	14.28	0.65	18	3.56	0.0963
Eczema	0.24	0.95	0.04	21	8.05	0.0072
Urticaria	0.64	2.06	0.18	23	1.09	0.4049
Chickenpox	0.54	1.25	0.21	43	3.93	0.0660
Meningococcal disease	N/A	0.17	0	34	34	< 0.0001
Jaundice	6.5	139.52	1.06	15	8.07	0.0074
HSP	3	11.73	0.97	28	7	0.0125

^a^OR’s were calculated as the number of students who got the NWS picture correct and WS incorrect divided by the number who got WS correct and NWS incorrect. OR < 1 can be interpreted as being better at diagnosing the WS picture, OR > 1 signifies better at diagnosing NWS picture

^b^N = From the number of participants who answered both picture types, the number of participants in each of the two opposing groups (WS correct + NWS incorrect and WS incorrect + NWS correct). Those who got both pictures correct or both incorrect were not included in the matched pairs odds ratios

^c^*P* value denotes the exact McNemar significance probability value for each condition

2.

## Appendix 2

See Table

**Table 3:** One sample Wilcoxon signed rank test, outcome as differences in confidence scores for each condition

	Z-score^a^	*P* value^b^	n^c^
Shingles	4.93	< 0.0001	141
Kawasaki disease	4.47	< 0.0001	116
Cellulitis	2.98	0.0028	136
Pityriasis versicolour	1.15	0.2507	106
Lyme disease	3.43	0.0005	103
Central cyanosis	− 1.73	0.0840	122
Eczema	6.95	< 0.0001	127
Urticaria	3.22	0.0011	115
Chickenpox	2.73	0.0062	128
Meningococcal disease	6.16	< 0.0001	108
Jaundice	− 4.51	< 0.0001	112
HSP	− 3.41	0.0005	98

^a^Null hypothesis was median = 0. Therefore, a Z score of > 0 indicates more confident with WS, < 0 more confident with NWS

^b^Exact probability for each Z score

^c^n = number of participants who submitted confidence ratings alongside diagnosis answers for both the WS and NWS picture of each condition, from the total 185 participants included in data analysis

3.
